# Inhibitory Effect of Lychee Seed Saponins on Apoptosis Induced by Aβ_25-35_ through Regulation of the Apoptotic and *NF-κB* Pathways in PC12 Cells

**DOI:** 10.3390/nu9040337

**Published:** 2017-03-29

**Authors:** Xiuling Wang, Hong Zhang, Jian Liu, Rong Chen, Yong Tang, Haixia Chen, Li Gu, Mao Li, Shousong Cao, Dalian Qin, Jianming Wu

**Affiliations:** 1 Department of Pharmacology, School of Pharmacy, Southwest Medical University, Luzhou 646000, China; wangxiulingbest@gmail.com (X.W.); m18989131787_1@163.com (H.Z.); belkn@sohu.com (J.L.); tangy1989@yeah.net (Y.T.); dna0805001@163.com (H.C.); tristan19810102@icloud.com (L.G.); batilimao@163.com (M.L.); shousongc@gmail.com (S.C.); 2 Department of Human Anatomy, School of Preclinical Medicine, Southwest Medical University, Luzhou 646000, China; 13558927205@163com; 3 Department of Human Anatomy, School of Preclinical Medicine, Sichuan Vocational College of Health and Rehabilitation, Zigong 643000, China; 4 Pharmacy Intravenous Admixture Services, Affiliated Hospital of Traditional Chinese Medicine, Southwest Medical University, Luzhou 646000, China

**Keywords:** Alzheimer’s disease, lychee seed saponins, Aβ_25-35_, PC12 cells, apoptosis, *NF-κB*

## Abstract

Neuronal apoptosis plays a critical role in the pathogenesis of Alzheimer’s disease (AD). Previous studies have shown that lychee seed saponins (LSS), isolated and extracted from traditional Chinese medicine lychee seeds, possess many beneficial activities including anti-oxidation, anti-diabetes, anti-AD, etc. In the present study, we established an in vitro neuronal apoptotic model of PC12 cells induced by Aβ_25-35_ and studied the effect of LSS on apoptosis by the methods of Hoechst 33342/propidium iodide (PI) fluorescence double staining, Annexin V/PI double staining, and terminal deoxynucleotidyl transferase (TdT)-mediated dUTP nick-end labeling (TUNEL). We also investigated the effects of LSS on mitochondria membrane potential, the expressions of Bcl-2 and Bax proteins, and the mRNA expression and the nuclear translocation of *NF-κBp65* in PC12 cells. The results showed that LSS markedly inhibited apoptosis, improved the mitochondria membrane potentials, upregulated the expression of Bcl-2 protein, downregulated the expression of Bax protein, and decreased the mRNA expression and nuclear translocation of *NF-κBp65* in PC12 cells. The study demonstrated that LSS significantly inhibited apoptosis induced by Aβ_25-35_ via regulation of the apoptotic and *NF-κB* pathways in PC12 cells. Therefore, LSS has the potential to be developed as a novel agent or nutrient supplement for the prevention and/or treatment of AD.

## 1. Introduction

Alzheimer’s disease (AD) is a common progressive neurodegenerative disorder that is characterized by the abnormal deposition of senile plaques (SPs) and neurofibrillary tangles (NFTs) owing to formations of amyloid-β protein (Aβ) and hyperphosphorylation of Tau protein in the brain [1]. A recent study has shown that apoptosis played a key role in the pathology of AD and the apoptotic ratio was obviously increased in the neurons of hippocampus of AD rats compared to those of normal rats [2]. Furthermore, excessive apoptosis of neurons was closely related to neuronal loss in the brains of AD rats [3,4]. Accumulating evidence suggests that the key pathological cause of AD is the neuronal injury and loss caused by neuronal apoptosis, although the accurate mechanism is still unknown.

Apoptosis is a biological process in which a sensitive cell proceeds towards programmed death upon receiving certainly stimuli. Neuronal apoptosis is influenced by multiple stimuli, such as activation of relevant enzymes [5], reactive oxygen species (ROS) [6], mitochondrial permeability transition [7], tumor necrosis factor-α (TNF-α) [8], Bcl-2, Bax, caspases [9], and so on. Aβ, as a hallmark in the pathogenesis of AD, can inhibit phosphatidylinositol 3 kinase (PI3K)/Akt to increase the expression of pro-apoptosis genes such as Bcl-2-associated death promoter (*BAD*), glycogen synthase kinase 3β (*GSK-3β*), and nuclear factor *(NF)-κB*, and lead to neuronal apoptosis [10,11]. Furthermore, the mitochondria, as the major site of adenosine triphosphate (ATP) generation, actively participated in the integration and implementation of different stimuli related to apoptosis [12]. In addition, distorted expressions of apoptosis-related proteins, including *Bak*, *Bad*, *Bax*, *Bcl-2*, *p53*, *Par-4*, and *Fas*, as well as the activation of caspases, have also been implicated in the pathogenesis of AD [13,14].

As a famous traditional Chinese medicine, lychee seed (Lizhi He in Chinese) has been recorded in the *Compendium of* *Materia Medica* (*Bencao Gangmu*) and exhibits many beneficial effects including anti-tumor [15], anti-oxidation [16], anti-virus [17,18], preventing liver injury [19], improving insulin resistance (IR) and sensitivity [20,21], and other pharmacological effects. Previous studies have shown that the chemical constituents of lychee seed are mainly saponins, organic acids, fatty acids, amino acids, sugars, and flavonoids [15]. Recently, studies have reported that the saponins from gypenoside, ginsenoside, and senegenin possess the effect of neuroprotection [22,23]. Our preliminary studies have shown that lychee seed saponins (LSS) significantly relieved the cognitive dysfunction in a rat model of type 2 diabetes, improving IR and inhibiting NF-κB-mediated apoptosis. Additionally, we found that LSS obviously decreased the formation of Aβ, tau protein, and advanced glycosylation end products (AGEs), as well as the release of mitochondrial cytochrome C, to execute its anti-apoptosis effect in the brains of AD rats [24]. In the present study, we investigated the anti-apoptotic effect of LSS by Hoechst 33342/propidium iodide (PI) fluorescence double staining, AnnexinV/PI double staining, and terminal dexynucleotidyl transferase (TdT)-mediated dUTP nick-end labeling (TUNEL) methods, mitochondria membrane potentials by Rhodamine 123 staining, the expressions of Bcl-2 and Bax proteins by Western blotting, and the mRNA expression and nuclear translocation of *NF-κBp65* by reverse transcription polymerase chain reaction (RT-PCR) or immunofluorescence analysis, respectively, in rat adrenal pheochromocytoma 12 (PC12) cells.

## 2. Materials and Methods

### 2.1. General Information

Aβ_25-35_ (Lot: Y-0044) was purchased from BIOSS Biotech Limited Company (Beijing, China). The Hoechst 33342/PI apoptotic assay kit was purchased from Sigma-Aldrich (St. Louis, MO, USA). The TUNEL apoptosis detection kit was purchased from BOSTER Biotech Limited Company (Wuhan, Hubei, China). The Annexin V-FITC/PI apoptotic detection kit was purchased from KeyGEN Biotech Corp., Ltd. (Nanjing, China). Rhodamine 123 was purchased from Yansheng biochemical reagent Co., Ltd. (Shanghai, China). The RT-PCR kit was purchased from TransGen Biotech Limited Company (Beijing, China). 4′,6-diamidino-2-phenylindole dihydrochloride (DAPI) was purchased from Sigma-Aldrich (Milan, Italy). Anti-NF-κBp65 antibody was purchased from Invitrogen (Carlsbad, CA, USA). The CO_2_ incubator and ultra-low temperature freezer were purchased from SANYO Electric Co., Ltd. (Moriguchi, Japan). The laser scanning confocal microscope was purchased from Leica DMIRB (Rueil Malmaison, France). The flow cytometer was purchased from Becton-Dickinson Labware (Franklin Lakes, NJ, USA). The gel imaging and analysis system ChemiDocXRS were purchased from Bio-Rad Laboratories, Inc. (Berkeley, CA, USA). The TC-512PCR amplification instrument was purchased from TECHNE (London, UK).

### 2.2. Cell Culture

PC12 cells were purchased from China Center for Type Culture Collection (CCTCC, Wuhan, Hubei, China). The cells were cultured in Dulbecco’s modified Eagle’s medium (DMEM) supplemented with 5% fetal bovine serum, 10% horse serum, penicillin (100 U/mL), and streptomycin (100 μg/mL) in a CO_2_ incubator (5% CO_2_, 37 °C) and renewed with new medium every 3–5 days. PC12 cell is a nervous-like cell derived from rat adrenal medulla pheochromocytoma and is a widely used cell line for the study of the nervous system in vitro [25].

### 2.3. Collection, Isolation, and Extraction of LSS

Lychee seed was purchased from the market in Luzhou (Luzhou, Sichuan, China) and authenticated by Professor Can Tang (Department of Chinese Materia Medica, School of Pharmacy, Southwest Medical University, Luzhou, Sichuan, China). One thousand grams of dried lychee seeds were ground and soaked in 1000 mL 70% ethanol overnight and extracted by percolation with 8000 mL 70% ethanol at a speed of 5 mL/min/kg. The solvents were evaporated under a vacuum and diluted to 5000 mL with distilled water and absorbed by D101 macroporous resins at speed of 6 mL/min. Finally, LSS was collected by elution with 800 mL 70% ethanol and concentrated to dryness by a rotary vacuum evaporator to yield 31.75 g of dried extract.

### 2.4. Establishment of an Apoptotic Cell Model of PC12 Cells Induced by Aβ25-35 and Drug Treatment In Vitro

First, PC12 cells were seeded at a density of 1.0 × 10^5^ cells/well on six-well plates (2.0 mL). After being cultured for 24 h, the culture medium was replaced with a serum-free DMEM medium. Next, the cells were treated with different concentrations (0.95, 1.90, 3.80, and 7.60 mg/L) of LSS for 1 h; serum-free DMEM medium was used as control. Then the cells were placed in a CO_2_ incubator and exposed to 20 μmol/L Aβ_25-35_ for 12 h to establish the cell model of AD. All experiments were performed in triplicate.

### 2.5. Apoptotic Analysis of PC12 Cells by Hoechst 33342/PI Fluorescence Double Staining

Apoptotic assay was processed by Hoechst 33342/PI fluorescence double staining as described previously [26]. Briefly, PC12 cells were treated with different concentrations of LSS (0.95, 1.90, 3.80, and 7.60 mg/L) or medium as control, then 10 μL (100 mg/L) Hoechst 33342 and 5 μL (1 mg/L) PI were added to the culture medium for 15 min at 37 °C, then washed with PBS and made cell resuspensions. The nuclear morphological changes of apoptotic cells were observed under fluorescence microscope and the percentage of apoptotic cells was calculated according to the ratio of apoptotic cells to total cells.

### 2.6. Apoptotic Analysis of PC12 Cell by Annexin V/PI Double Staining

To confirm early apoptotic cells, flow cytometer were used through FITC-conjugated Annexin V and PI double staining as described previously [27]. PC12 cells were treated with different concentrations of LSS (0.95, 1.90, 3.80, and 7.60 mg/L) or medium as control and harvested with 0.25% trypsin, washed twice with PBS, resuspended in buffer and incubated with 5 μL AnnexinV-FITC and 5 μL PI for 15 min in the dark at 37 °C. Then the specific fluorescence of 12,000 cells was observed on a FACSCalibur (BD) system within 1 h. Data were analyzed according to the apoptotic ratio.

### 2.7. Apoptotic Analysis of PC12 Cells by TUNEL

The apoptotic studies were also carried out with TUNEL analyses as previous described [28]. PC12 cells were treated with different concentrations of LSS (0.95, 1.90, 3.80, and 7.60 mg/L) or medium as control and evaluated by TUNEL immunohistochemical assay according to the manufacturer’s instruction. The apoptotic cells were showed brown colors in the nuclei of cells. The apoptotic ratio was calculated as the percentage of apoptotic cells among 100 cells by GD-10.0 image analysis system.

### 2.8. Mitochondrial Membrane Potential Examination in PC12 Cells

Rhodamine 123 was used as a probe to detect the changes of mitochondria membrane potentials (ΔΨmt). Briefly, PC12 cells were washed twice with PBS and incubated with Rhodamine 123 at the concentration 1 μmol/L for 30 min at 37 °C in a CO_2_ incubator. After washing twice with PBS, the cells were observed under a fluorescence microscope and images were recorded.

### 2.9. Western Blotting Analysis of the Protein Expressions of Bcl-2 and Bax in PC12 Cells 

PC12 cells were seeded at a density of 1.0 × 10^5^ cells/well on six-well plates (2.0 mL), after being cultured for 24 h, the culture medium was replaced with serum-free DMEM medium. Then, the cells were randomly divided into six groups, rinsed with a serum-free DMEM medium, and treated with serum-free medium (vehicle as control in two groups) or LSS (0.95, 1.90, 3.80, and 7.60 mg/L) for 1 h. Next, the cells in one control group and four groups of LSS were exposed to 20 μmol/L Aβ_25-35_ and another control group was treated with a medium for 13 h without Aβ_25-35_.

After being cultured for 12 h, the culture medium was withdrawn and the cells were washed with cold PBS for harvest. The cell pellets were disrupted in a cell lysis buffer, 0.5% NP-40, 50 mmol/L Tris-HCl, 120 mmol/L NaCl, 1 mmol/L EDTA, 0.1 mmol/L Na_3_VO_4_, 1 mmol/L NaF, 1 mmol/L PMSF, and 1 μg/mL leupeptin, pH 7.5), then the lysates were centrifuged at 9600× *g* for 15 min at 4 °C. The protein concentrations were determined using the bicinchoninic acid (BCA) method, after which equal amounts of protein (30 μg) were electrophoresed on 10% density of SDS-acrylamide gels. Following electrophoresis, the proteins were transferred from the gel to a nitrocellulose membrane using an electric transfer system. Non-specific binding was blocked with 5% skim milk in tris-buffered saline with tween (TBST) buffer, 5 mmol/L Tris-HCl, 136 mmol/L NaCl, and 0.1% Tween-20, pH 7.6) for 1 h. The blots were incubated with antibodies against Bcl-2 (1:1000), Bax (1:1000) or β-actin (1:800) overnight at 4 °C and were washed three times with 1× TBST. Then, the blots were incubated for 1 h at room temperature with a 1:1000 dilution of horseradish peroxidase-labeled anti-rabbit or anti-mouse IgG and washed three times with 1× TBST, the membranes were developed by incubation within the ECL Western detection reagents. The specific protein bands were visualized and analyzed with a ChemiDoc image analyzer (Bio-Rad, Hercules, CA, USA).

### 2.10. RNA Isolation and Analysis of NF-κBp65 mRNA Expression in PC12 Cells by RT-PCR

Total RNA from PC12 cells was extracted using TRIzol reagent. The purity and concentration of mRNA were determined by sepharose gel and UV spectrophotometer, respectively. cDNA was synthesized through the reverse transcription of 1 μg mRNA using Taq PCR Master Mix. The primer sequences for NF-κBp65 and glyceraldehyde-3-phosphate dehydrogenase (GAPDH) were as follows: rat NF-κBp65 5′-ACCAAAGACCCACCTCACC-3′ (forward) and 5’-CGCATTCAAGTCATAGTCCC-3′ (reverse); rat GAPDH 5′-CCTCAAGATTGTCAGCAAT-3′ (forward) and 5′-CCATCCACAGTCTTCTGAGT-3′ (reverse), respectively. The reaction of PCR was conducted according to the manufacturer's protocol. The products of PCR underwent 2% agarose gel electrophoresis with a weight marker of DL500 DNA molecular. Gel imaging and analysis system were conducted for examination and quantitative analyses. GAPDH was used as the control.

### 2.11. Nuclear Translocation of NF-κBp65 in PC12 Cells by Immunofluorescence Analysis

Nuclear translocation of NF-κBp65 was studied by immunofluorescence analysis. PC12 cells were fixed in 4% paraformaldehyde for 30 min at 4 °C, then washed with 0.1% PBS and incubated with the primary antibody (1:50) overnight at 4 °C. The cells were washed with 0.1% PBS and incubated with anti-NF-κBp65 antibody (1:400) for 1 h and stained with 4′, 6-diamidino-2-phenylindole dihydrochloride (DAPI). The images were recorded and assessed under fluorescence microscopy.

### 2.12. Statistical Analysis

All data are expressed as mean ± standard deviation (SD). Statistical differences of the data among the means of two or more groups were analyzed by one-way univariate analysis of variance (ANOVA). A difference at *p* < 0.05 was considered to be statistically significant (marked as *). The higher significance level was set at *p* < 0.01 (marked as **).

## 3. Results

### 3.1. Effects of LSS on the Aβ_25-35_-Induced Apoptosis in PC12 Cells by Hoechst 33342/PI Fluorescence Double Staining, Annexin V/PI Double Staining, and TUNEL Analyses

**Figure 1:**
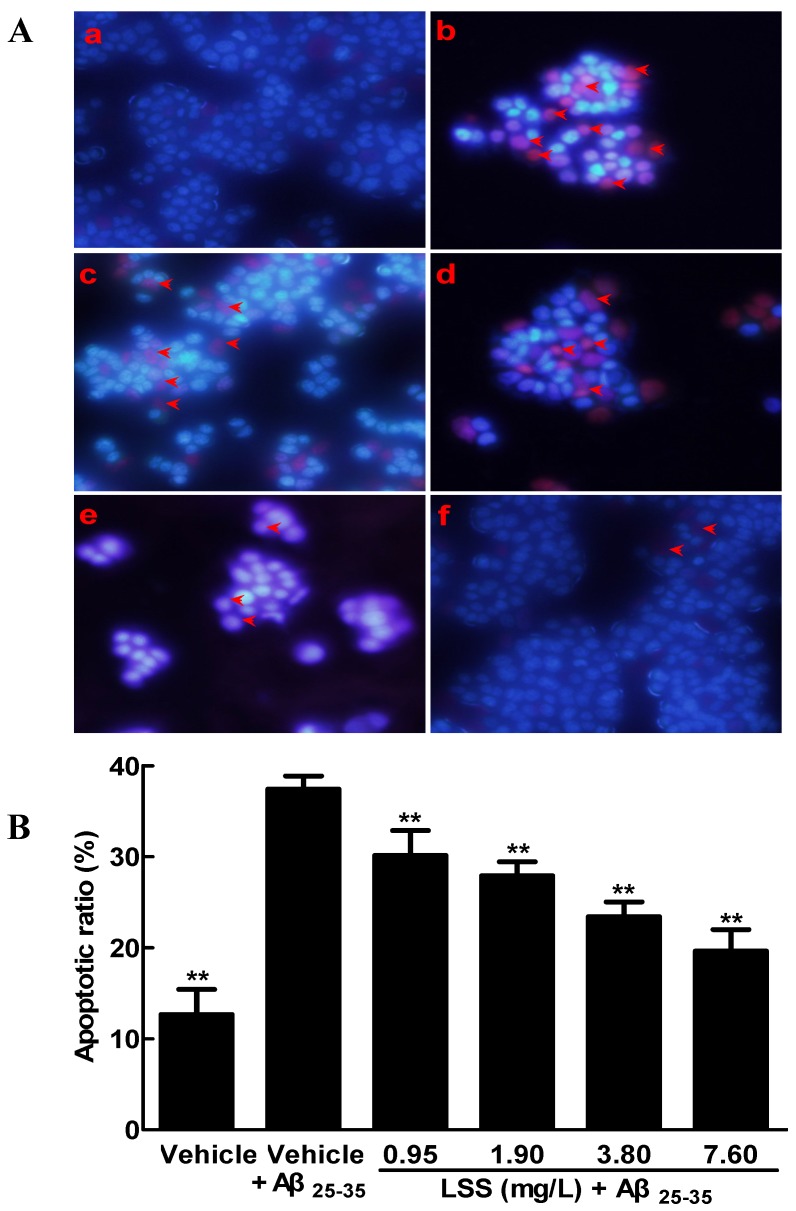
Effect of LSS on apoptosis induced by Aβ_25-35_ (20 μmol/L) in PC12 cells with Hoechst 33342/PI staining under fluorescence microscope (400×). (**A**) Representative pictures of PC12 cells treated with or without LSS; (**Aa**) cells were treated with medium for 13 h without Aβ_25-35_; (**Ab**) cells were treated with medium for 1 h and followed by Aβ_25-35_ for 12 h; (**Ac**) cells were treated with LSS 0.95 mg/L for 1 h and followed by Aβ_25-35_ for 12 h; (**Ad**) cells were treated with LSS 1.90 mg/L for 1 h and followed by Aβ_25-35_ for 12 h; (**Ae**) cells were treated with LSS 3.80 mg/L for 1 h and followed by Aβ_25-35_ for 12 h; (**Af**) cells were treated with LSS 7.60 mg/L for 1 h and followed by Aβ_25-35_ for 12 h. The red arrows indicate apoptotic cells; (**B**) Summarized results of apoptotic ratio. The results are representative of at least three independent experiments run in triplicate and expressed as mean ± SD. ** *p* < 0.01 vs. cells treated with vehicle and Aβ_25-35_.

The key pathogenesis of AD is the neuronal loss caused by apoptosis. Therefore, we studied the effect of LSS on apoptosis induced by Aβ_25-35_ in PC12 cells through Hoechst 33342/PI double staining under a fluorescence microscope. The representative morphological pictures of PC12 cells and the cell apoptotic ratio are illustrated in Figure 1. The picture in Figure 1(Aa) shows the characteristic preserved normal features of PC12 cells in the control cells without Aβ_25-35_ treatment, which the nuclei of cells appear blue fluorescence. However, higher levels of apoptotic cells with condensation of nuclear chromatin and fragmentation were detected in the cells treated with Aβ_25-35_ (20 μmol/L) for 12 h (Figure 1(Ab)). Interestingly, the numbers of apoptotic cells were obviously decreased after LSS (0.95–7.60 mg/L) treatment (Figure 1(Ac–f)). The summarized results of the apoptotic ratio of cells are shown in Figure 1B and indicate that Aβ_25-35_ treatment significantly increased apoptosis compared to control cells treated with vehicle (*p <* 0.01), but LSS significantly inhibited Aβ_25-35_-induced apoptosis compared to vehicle treatment (*p <* 0.01) in PC12 cells. 

**Figure 2:**
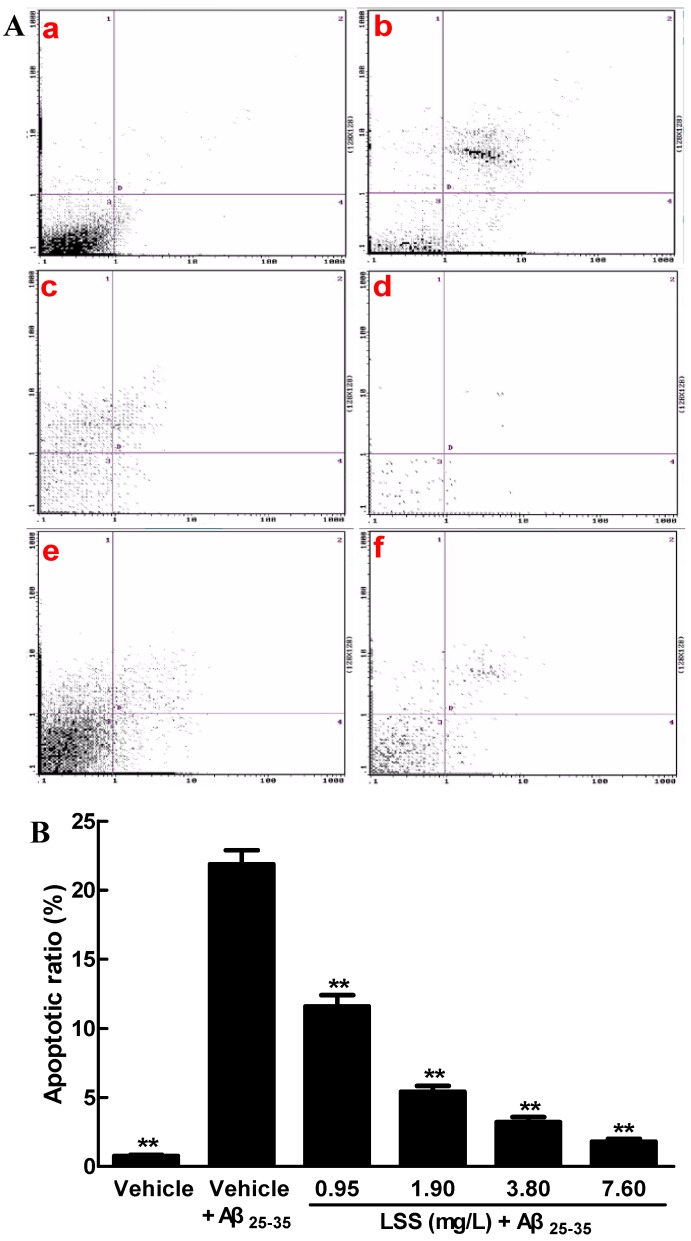
Effect of LSS on apoptosis (**A**) and apoptotic ratio (**B**) induced by Aβ_25-35_ (20 μmol/L) in PC12 cells with Annexin V/PI staining by flow cytometric analysis. (**Aa**) Cells were treated with medium for 13 h without Aβ_25-35_; (**Ab**) cells were treated with medium for 1 h and followed by Aβ_25-35_ for 12 h; (**Ac**) cells were treated with LSS 0.95 mg/L for 1 h and followed by Aβ_25-35_ for 12 h; (**Ad**) cells were treated with LSS 1.90 mg/L for 1 h and followed by Aβ_25-35_ for 12 h; (**Ae**) cells were treated with LSS 3.80 mg/L for 1 h and followed by Aβ_25-35_ for 12 h; (**Af**) cells were treated with LSS 7.60 mg/L for 1 h and followed by Aβ_25-35_ for 12 h; (**B**) Summarized results of apoptotic ratio. The results are representative of at least three independent experiments run in triplicate and expressed as mean ± SD. ** *p* < 0.01 vs. the cells treated with vehicle and Aβ_25-35_.

Next, we investigated the effect of LSS on apoptosis induced by Aβ_25-35_ in PC12 cells with Annexin V/PI double staining by flow cytometric analysis and the results are shown in Figure 2. The data showed that Aβ_25-35_ (20 μmol/L) treatment for 12 h markedly increased apoptosis in PC12 cells to 21.89% ± 1.01% (*p* < 0.01) compared to 0.78% ± 0.07% in control cells treated with vehicle (Figure 2(Aa,b)). However, LSS treatments (0.95–7.60 mg/L) significantly decreased apoptotic cells (*p* < 0.01) in PC12 cells exposed to Aβ_25-35_ (20 μmol/L) to 11.59% ± 0.81%, 5.41% ± 0.43%, 3.20% ± 0.37%, and 1.81% ± 0.19%, respectively (Figure 2(Ac–f)). The summarized results also clearly demonstrated that LSS effectively inhibited apoptosis in a concentration-dependent manner in PC12 cells with Annexin V/PI staining by flow cytometric analysis (Figure 2B).

**Figure 3:**
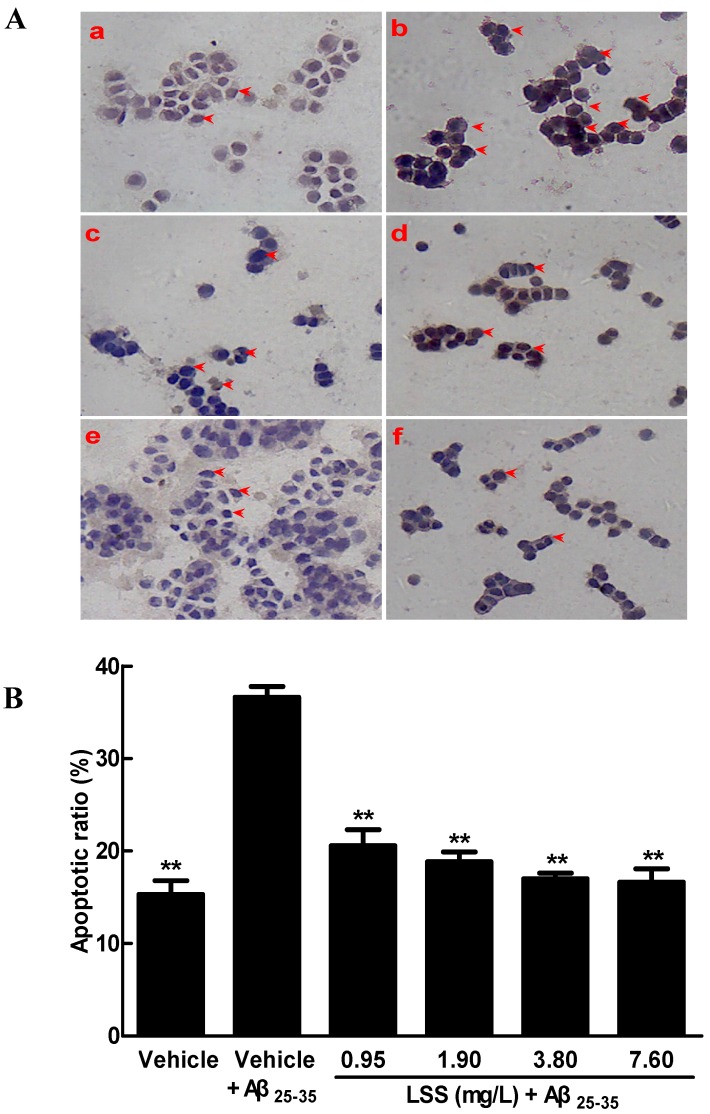
Effect of LSS on apoptosis (**A**) and apoptotic ratio (**B**) induced by Aβ_25-35_ (20 μmol/L) in PC12 cells with TUNEL analysis (×400). (**Aa**) Cells were treated with medium for 13 h without Aβ_25-35_ ; (**Ab**) cells were treated with medium for 1 h and followed by Aβ_25-35_ for 12 h; (**Ac**) cells were treated with LSS 0.95 mg/L for 1 h and followed by Aβ_25-35_ for 12 h; (**Ad**) cells were treated with LSS 1.90 mg/L for 1 h and followed by Aβ_25-35_ for 12 h; (**Ae**) cells were treated with LSS 3.80 mg/L for 1 h and followed by Aβ_25-35_ for 12 h; (**Af**) cells were treated with LSS 7.60 mg/L for 1 h and followed by Aβ_25-35_ for 12 h. The red arrows indicate apoptotic cells; (**B**) Summarized results of apoptotic ratio. The results are representative of at least three independent experiments run in triplicate and expressed as mean ± SD. ** *p* < 0.01 vs. the cells treated with vehicle and Aβ_25-35_.

Finally, we further studied the apoptosis of PC12 cells by TUNEL immunohistochemical assay. The representative photomicrographs are shown in Figure 3, a few spontaneous apoptotic cells are clearly observed, with brown appearance in the control cells treated with a vehicle (Figure 3(Aa)). Higher levels of apoptosis were detected in the cells treated with 20 μmol/L Aβ_25-35_ (Figure 3(Ab)), and the apoptotic ratio obviously increased compared to that of the control cells (*p <* 0.01). However, the numbers of apoptotic cells were significantly decreased (*p <* 0.01) after LSS treatment (Figure 3Ac–f) compared to after vehicle treatment. The summarized results are illustrated in Figure 3B and the data also indicate that LSS can markedly inhibit the apoptosis induced by Aβ_25-35_ in PC12 cells. The results are consistent with those of the Hoechst33342/PI fluorescence double staining and AnnexinV/PI double staining methods. 

### 3.2. Effect of LSS on Mitochondrial Membrane Potential in PC12 Cells Exposed to Aβ_25-35_ with Rhodamine 123 Staining

**Figure 4:**
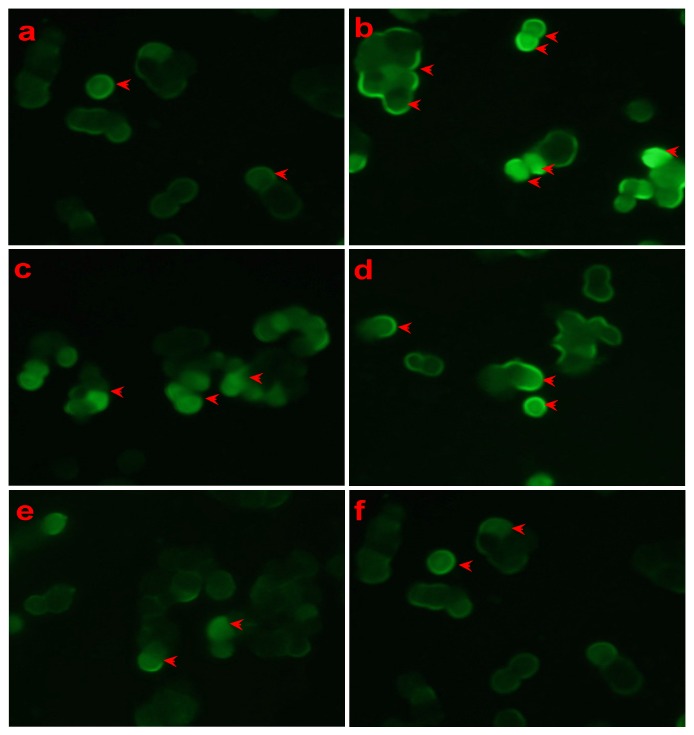
Effect of LSS on mitochondria membrane potential in PC12 cells treated with or without Aβ_25-35_ (20 μmol/L) with rhodamine 123-staining (400×). (**a**) Cells were treated with medium for 13 h without Aβ_25-35_; (**b**) cells were treated with medium for 1 h and followed by Aβ_25-35_ for 12 h; (**c**) cells were treated with LSS 0.95 mg/L for 1 h and followed by Aβ_25-35_ for 12 h; (**d**) cells were treated with LSS 1.90 mg/L for 1 h and followed by Aβ_25-35_ for 12 h; (**e**) cells were treated with LSS 3.80 mg/L for 1 h and followed by Aβ_25-35_ for 12 h; (**f**) cells were treated with LSS 7.60 mg/L for 1 h and followed by Aβ_25-35_ for 12 h. The red arrows indicate apoptotic cells. The results are representative of at least three independent experiments run in triplicate.

Mitochondria play a key role in the progression of apoptosis. Therefore we examined the effect of LSS on mitochondrial membrane potential in PC12 cells with or without exposure to Aβ_25-35_ (20 μmol/L) for 12 h with rhodamine 123 staining and the results of the representative photomicrographs are shown in Figure 4. The fluorescence intensity of PC12 cells induced by Aβ_25-35_ was obviously increased (*p* < 0.01) compared to that of the control cells treated with a medium (Figure 4a,b). However, a significantly decrease in fluorescence intensity was observed after PC12 cells were treated with LSS at 0.95, 1.90 3.80 and 7.60 mg/L for 12 h compared to the control cells treated with medium (Figure 4c–f). The results indicate that the mitochondrial membrane potentials of P12 cells were decreased by LSS compared to vehicle treatment.

### 3.3. Effects of LSS on the Protein Expressions of Bcl-2 and Bax in PC12 Cells Induced by Aβ_25-35_

**Figure 5:**
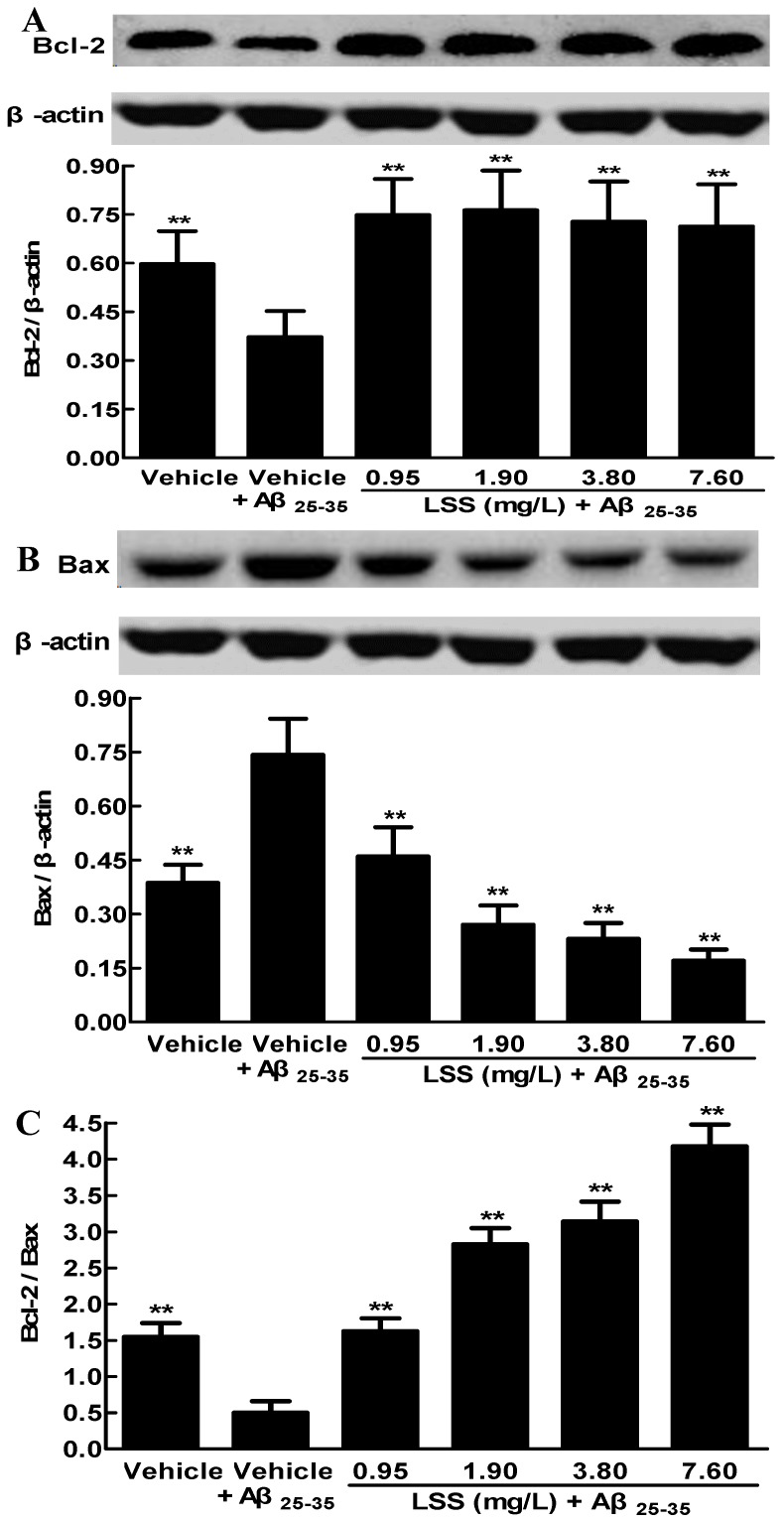
Effects of LSS on the protein expressions of Bcl-2 (**A**), Bax (**B**), and the ratio of Bcl-2/Bax (**C**) in PC12 cells with or without exposure to 20 μmol/L Aβ_25-35_ for 12 h. The results are representative of at least three independent experiments run in triplicate and expressed as mean ± SD. ** *p* < 0.01 vs. the cells treated with Aβ_25-35_ and vehicle.

The protein expressions of Bcl-2 and Bax are crucial for the mitochondria-mediated apoptosis. Therefore, we examined the protein expressions of Bcl-2 and Bax in PC12 cells by Western blotting analysis. The results showed that LSS can inhibit the apoptosis significantly in PC12 cells through regulation the protein expressions of Bcl-2 and Bax. In which the protein expression of Bcl-2 was downregulated, while that of Bax was significantly upregulated exposed to 20 μmol/L Aβ_25-35_ for 12 h compared to cells treated with a vehicle (*p* < 0.01) (Figure 5A,B). However, LSS at 0.95–7.60 mg/L significantly upregulated the protein expression of *Bcl-2* compared to vehicle treatment (*p* < 0.01) in PC12 cells, which decreased by Aβ_25-35_ (Figure 5A). Furthermore, LSS also significantly downregulated the overexpression of Bax induced by Aβ_25-35_ in a concentration-dependent manner compared to vehicle treatment (*p* < 0.01) in PC12 cells (Figure 5B). Therefore, LSS increases the ratio of Bcl-2/Bax in PC12 cells treated with Aβ_25-35_ in a concentration-dependent manner (*p* < 0.01) (Figure 5C). The results demonstrate that the regulation of the expressions of Bcl-2 and Bax by LSS is an important mechanism of protection of PC12 cells from Aβ_25-35_ induced apoptosis and cell damage.

### 3.4. Effects of LSS on mRNA Expression and Nuclear Translocation of NF-κBp65 in PC12 Cells Treated with Aβ_25-35_

**Figure 6:**
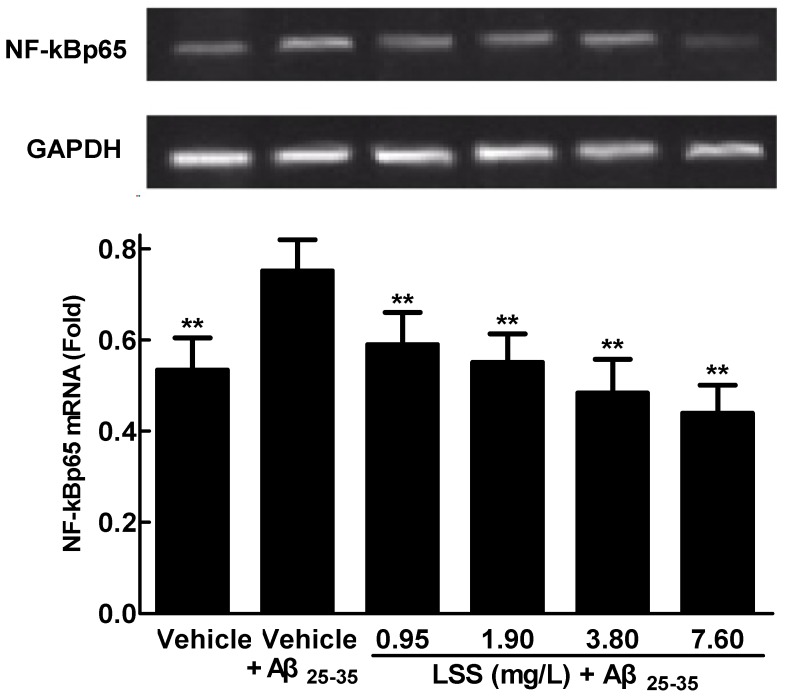
Effect of LSS on the mRNA expression of *NF-κBp65* in PC12 cells treated with Aβ_25-35_ (20 μmol/L). The results are representative of at least three independent experiments run in triplicate and expressed as mean ± SD. ** *p* < 0.01 vs. the cells treated with Aβ_25-35_ and vehicle.

We speculated that the effect of LSS on anti-apoptosis may be involved in regulation of the mRNA expression of *NF-κBp65* in PC12 cells. Therefore, the expression levels of *NF-κBp65* and GAPDH were analyzed by RT-PCR in PC12 cells with or without exposure to 20 μmol/L Aβ_25-35_ for 12 h and the results are shown in Figure 6. The data reveal that the mRNA expression of *NF-κBp65* significantly increased in PC12 cells by Aβ_25-35_ compared to the control cells treated with a vehicle (*p* < 0.01). However, LSS (0.95–7.60 mg/L) significantly decreased the mRNA expression of *NF-κBp65* (*p* < 0.01) in a concentration-dependent manner in PC12 cells treated with Aβ_25-35_.

**Figure 7:**
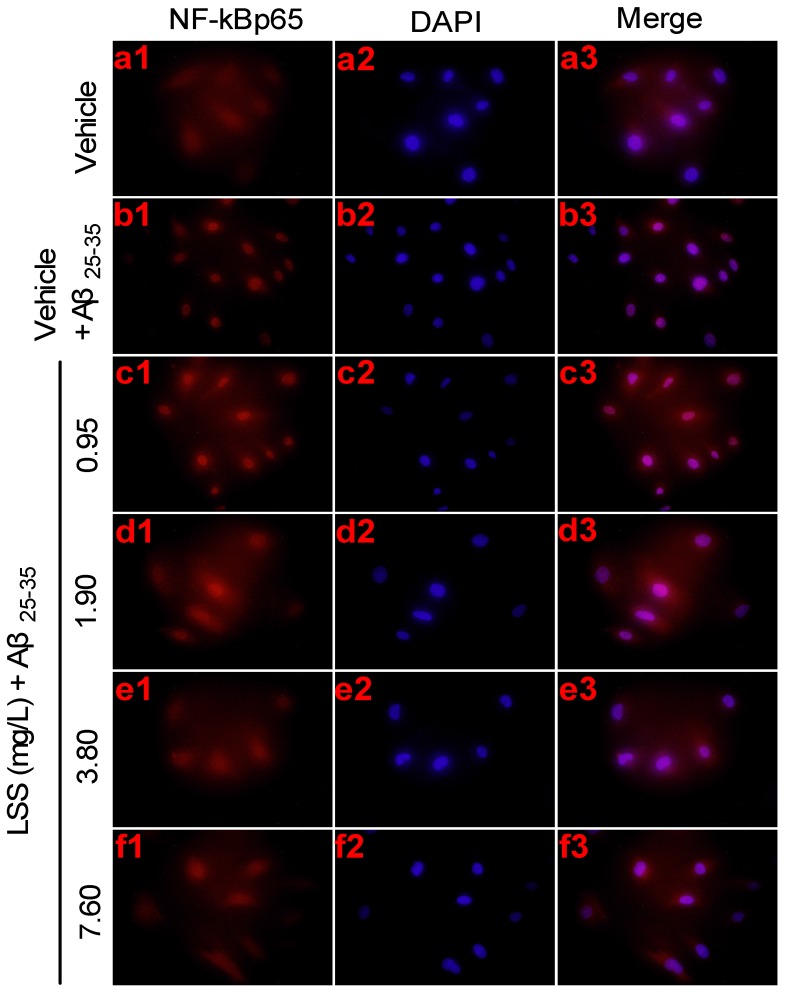
Effect of LSS on nuclear translocation of *NF-κBp65* in PC12 cells treated with or without 20 μmol/L Aβ_25-35_ for 12 h (400×). (**a**) Control cells were treated with medium for 13 h without Aβ_25-35_; (**b**) cells were treated with medium for 1 h and followed by 20 μmol/L Aβ_25-35_ for 12 h; (**c**) cells were treated with LSS 0.95 mg/L for 1 h and following by 20 μmol/L Aβ_25-35_ for 12 h; (**d**) cells were treated with LSS 1.90 mg/L for 1 h and followed by 20 μmol/L Aβ_25-35_ for 12 h; (**e**) cells were treated with LSS 3.80 mg/L for 1 h and followed by 20 μmol/L Aβ_25-35_ for 12 h; (**f**) cells were treated with LSS 7.60 mg/L for 1 h and followed by 20 μmol/L Aβ_25-35_ for 12 h. Numbers preceding letters represent the following: 1. red channel (NF-κB); 2. blue channel (DAPI); and 3. combined figure of red and blue channels. The results are representative of at least three independent experiments run in triplicate.

To investigate the effect of LSS on signaling downstream of *NF-κBp65* activation induced by Aβ_25-35_, we further studied the nuclear translocation of *NF-κBp65* in PC12 cells treated with or without 20 μmol/L Aβ_25-35_ by immunofluorescence analysis; and the results are shown in Figure 7. As shown by the representative pictures, *NF-κBp65* was mainly localized in the cytosolic compartment in the control cells treated with vehicle (Figure 7a). While Aβ_25-35_ strongly induced nuclear translocation of *NF-κBp65* (Figure 7b), LSS at 0.95, 1.90, 3.80, and 7.6 mg/L significantly decreased (*p* < 0.01) nuclear translocation of *NF-κBp65* induced by Aβ_25-35_ in a concentration-dependent manner (Figure 7c–f). The results indicate that Aβ_25-35_ markedly elevated mRNA expression and nuclear translocation of *NF-κBp65* and LSS significantly attenuated their increases in PC12 cells.

## 4. Discussion

The present study aimed to investigate the effects of LSS on apoptosis using three different analysis methods(Hoechst 33342/propidium iodide fluorescence double staining, Annexin V/PI double staining, and terminal deoxynucleotidyl transferase (TdT)-mediated dUTP nick-end labeling), mitochondrial function, protein expressions of *Bcl-2* and *Bax*, and mRNA expression and nuclear translocation of the *NF-κB* signaling pathway-associated apoptotic gene (*p65*) in PC12 cells treated with 20 μmol/L Aβ_25-35_ in vitro. Our results indicate that LSS significantly protects from apoptotic injury to the cells induced by Aβ_25-35_ (Figure 1, Figure 2 and Figure 3), decreases mitochondria membrane potentials (Figure 4), upregulates the expression of Bcl-2 protein, and downregulates the expressions of *Bax* protein and the ratio of *Bcl-2/Bax* (Figure 5), and inhibits the mRNA expression and nuclear translocation of *NF-κBp65* (Figure 6 and Figure 7) in Aβ_25-35_-treated PC12 cells. The results suggest that LSS can inhibit Aβ_25-35_-induced apoptosis through improvement of mitochondrial function and suppression of the *NF-κB* signaling pathway. Therefore, our findings suggest that the effect of LSS against AD may be involved with apoptotic and *NF-κB* signaling pathways in PC12 cells.

Aβ, as one of the main toxic peptides and a vital biomarker of neuronal apoptosis for AD, is derived from amyloid-β protein precursor (APP) processing by β-and γ-secretase through the amyloid cascade pathway [29]. Accumulating evidence suggests that Aβ contributes to AD pathogenesis and progress by various mechanisms including DNA fragmentation [30], Tau protein phosphorylation [31], mitochondrial dysfunction [32], and apoptosis induction. Various preclinical studies have provided evidence that Aβ_25-35_ and Aβ_1–40_ can induce neuronal apoptosis in PC12 and SH-SY5Y cells in vitro [33,34] and the hippocampus of brain in rats in vivo [35]. The underlying mechanism involved in neuronal apoptosis may be associated with multiple pathways, including the action of catabolic enzymes [36], release of ROS [36], disruption of mitochondria [37], and activation of the related apoptotic signal pathway and *NF-κB* pathway [38]. Numerous studies have indicated that the mitochondria play a vital role in cellular apoptosis [39,40]. The release of mitochondrial cytochrome c is an early common event in apoptosis [41]. Furthermore, the decrease of mitochondrial membrane potential in cell and apoptosis-related factors are key events in initiating the cascade reaction leading to cell apoptosis [42]. Aβ initiated mitochondrial oxidative stress and increased mitochondrial membrane permeability and cytochrome c release [42] to trigger the caspase cascade [43] and DNA degradation [44], leading to apoptosis. In addition, it is well known that cell survival is initiated by various factors including *NF-κB* and *Akt*, involved with *PI3K/Akt* signal transduction pathway [45]. Classical NF-κB, as a nuclear transcription factor, is a heterodimer composed of *p50/p65* subunits [46]. It is well established that activated forms of *PI3K/Akt* initiated *NF-κB* transcriptional activity, mainly through signaling pathway that stimulated the transactivation domain of the p65 subunit [47].

PC12 cells are derived from adrenal medulla pheochromocytoma in rats and the cells can be induced by some nerve growth factors (NGF) to differentiate into nervous-like cells with similar characteristics to neurons in physiological and/or biochemical aspects [25,48]. The cells with or without NGF are widely used to study various aspects on the field of nervous system in vitro [25,34,49]. In a previous study, we evaluated the cytotoxicity of LSS in PC12 cells to determine the maximum safe concentration and the effective concentration of LSS in protection from Aβ_25-35_ induced cytotoxicity [50,51].

**Figure 8:**
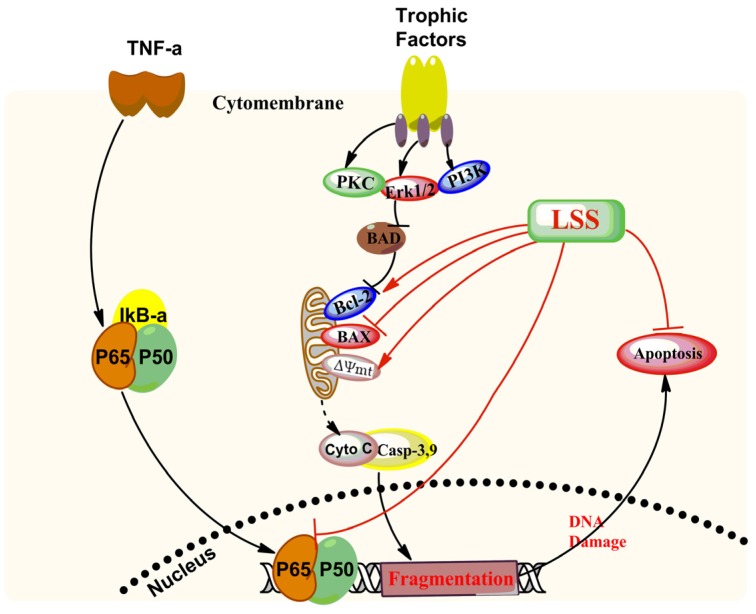
Proposed scheme of possible mechanisms for LSS inhibition of apoptosis induced by Aβ_25-35_ through regulation of the apoptotic and NF-κB pathways in PC12 cells.

Our previous studies have found that LSS can decrease the formation of Aβ and mitochondrial cytochrome C release, thereby inhibiting neuronal apoptosis in the brain of AD rats [51]. In the present study, we found that the anti-apoptotic effect of LSS in PC12 cells treated with Aβ_25-35_ may be associated with mitochondrial membrane function and the *NF-κB* signaling pathway. The proposed possible mechanisms for LSS effects are summarized in Figure 8. However, it is still unclear whether LSS can affect other signaling pathways such as protein kinase C (*PKC*), *PI3K/Akt*, and *Jak/stat* pathways due to the complexity of the molecular mechanisms in the pathogenesis of AD. Therefore, our further investigation of the molecular mechanisms associated with the effect of LSS on neuronal protection in AD should include the upstream regulation of *PI3K/Akt* and apoptotic pathways, as well as the protein expression and phosphorylation of *P65* and *P50* related to the NF-κB pathway.

## 5. Conclusions

The present study demonstrated that LSS significantly inhibited apoptosis in PC12 cells induced by Aβ_25-35_. The possible mechanisms may be related to improvement of mitochondrial function via inhibition of the mitochondria membrane permeability, upregulating the expressions of Bcl-2 protein, downregulating the expression of Bax protein, and decreasing the mRNA expression and nuclear translocation of NF-κBp65 in PC12 cells exposed to 20 μmol/L Aβ_25-35_ for 12 h. Therefore, LSS has the potential to be developed as a novel agent or nutrient supplement for the prevention and/or treatment of AD clinically.
